# Editorial: AntibodyPlus therapeutics in oncology: innovative mechanisms, therapeutic strategies, and clinical advances

**DOI:** 10.3389/fonc.2026.1896596

**Published:** 2026-06-15

**Authors:** Yuanzhi Chen, Pengwei Ren, Heyu Chen, Maozhou He

**Affiliations:** 1 School of Medicine, Huaqiao University, Xiamen, China; 2 Department of Surgery, Yale University School of Medicine, New Haven, CT, United States; 3 AstraZeneca, Gaithersburg, MD, United States; 4 School of Pharmaceutical Science and Technology, Hangzhou Institute for Advanced Study, University of Chinese Academy of Sciences, Hangzhou, China

**Keywords:** antibody engineering, antibody therapy, AntibodyPlus, cancer immunotherapy, tumor microenvironment modulation

In recent years, antibody-based therapeutics have undergone a profound transformation, evolving from conventional monoclonal antibodies into a diverse class of multifunctional platforms collectively referred to as “AntibodyPlus.” These advanced modalities—including antibody-drug conjugates (ADCs), bispecific and multispecific antibodies, and immune-modulating constructs—are reshaping the paradigm of cancer treatment by integrating targeting specificity with enhanced therapeutic functionality. As editors of this Research Topic, we are pleased to present a collection of studies that highlight the rapid progress and emerging opportunities in this field. A total of 23 manuscripts were submitted, of which 11 were accepted after rigorous peer review and ultimately published, reflecting both the high level of interest and the stringent selection standards applied to this Topic. The contributions gathered here span fundamental mechanisms, technological innovations, and clinical translation, offering a comprehensive overview of the current landscape of AntibodyPlus therapeutics in oncology.

Among the diverse engineering strategies presented, HER2-targeted biparatopic and nanobody-based approaches illustrate efforts to overcome therapeutic resistance through improved receptor engagement. Liu et al., “*Biparatopic HER2-targeted nanobody binder synergizes with trastuzumab to overcome resistance in tumor cells*,” demonstrated that biparatopic nanobody constructs enhance receptor binding and restore sensitivity in resistant HER2-positive tumors. Building on this, Liu et al., “*Engineering HER2-targeted biparatopic antibodies to enhance receptor internalization and antitumor activity*,” further showed that optimized designs promote receptor internalization and improve therapeutic efficacy. Together, these studies highlight the importance of antibody structural design in modulating receptor dynamics and overcoming resistance.

Beyond biparatopic strategies, alternative antibody formats are also explored. Wang et al., “*Harnessing IgM for solid tumor therapy: biology, engineering advances, and translational challenges*,” reviewed the unique advantages of IgM antibodies, particularly their multivalency and high avidity, while addressing key challenges in manufacturing and clinical translation. This work underscores the potential of expanding beyond traditional IgG formats to enhance therapeutic performance.

In parallel, several studies focus on targeting immune regulatory pathways. Campos et al., “*A fully human IgG1 antibody targeting MICA α1 domain inhibits interaction with NKG2D and activates immune effector functions against MICA-expressing cells*,” developed a monoclonal antibody that both blocks the MICA–NKG2D interaction and activates Fc-mediated effector functions such as ADCC, ADCP, and complement activation. By targeting a low-polymorphic region, this approach enables broad allele coverage and provides a rational strategy to overcome antigen heterogeneity.

Advances in antibody-based drug delivery are also prominently featured. Guo et al., “*Clinical applications of antibody-drug conjugates in advanced non-small cell lung cancer*,” analyzed the development of ADCs in NSCLC, highlighting how optimized linker–payload systems and tumor-specific targeting can improve efficacy while reducing toxicity. The study also emphasizes ongoing challenges, including tumor heterogeneity, resistance, and the need for biomarker-guided patient selection.

Innovative platforms for localized antibody delivery are further explored by Kang et al., “*Engineering antibody-armed oncolytic viruses: design strategies, synergistic mechanisms, and clinical translation*,” which described oncolytic viruses engineered to express therapeutic antibodies within the tumor microenvironment. This strategy enables spatially restricted delivery and sustained intratumoral expression, addressing limitations of systemic therapy and enhancing the synergy between viral oncolysis and immune modulation.

Targeting immunosuppressive signaling pathways represents another important direction. Kang et al., “*Antibody targeting of the TNF–TNFR2 axis to overcome tumor immune resistance*,” identified TNFR2 as a central regulator of immunosuppressive networks involving Tregs, MDSCs, CAFs, and tumor cells. The study highlights the development of diverse antibody-based strategies—including monoclonal antibodies, ADCs, and bispecific formats—to selectively disrupt this axis and restore antitumor immunity.

From a diagnostic and translational perspective, Li et al., “*Development and characterization of a novel B7-H3 rabbit monoclonal antibody for glioma diagnosis*,” reported a high-specificity antibody targeting B7-H3, demonstrating strong performance across multiple platforms. The high prevalence of B7-H3 expression in glioma further supports its value as both a biomarker and a companion diagnostic.

Clinical application is further illustrated by Shao et al., “*Clinical trials of bispecific antibody therapy for colorectal cancer: advanced and next steps*,” which analyzed the global landscape of bispecific antibody trials in colorectal cancer. The study reveals rapid clinical expansion and increasing focus on dual-target strategies, highlighting their potential in overcoming immune escape and improving therapeutic precision.

At the mechanistic level, Choi et al., “*A novel anti-CD20, concabody, enhances immunotherapy efficacy by targeting MPZL1 and augmenting antibody-induced cell death*,” introduced a novel antibody–lectin fusion format termed “concabody.” By enhancing lysosome-mediated cell death alongside ADCC, this strategy provides new insight into antibody-induced cytotoxicity while improving therapeutic efficacy and specificity.

Finally, Sofianidi et al., “*Sacituzumab tirumotecan (sac-TMT/MK-2870/SKB264): a novel antibody–drug conjugate in breast cancer*,” reviewed a next-generation TROP2-targeting ADC with a novel linker and payload. Clinical data demonstrate promising efficacy and manageable toxicity, particularly in triple-negative breast cancer, highlighting the impact of incremental design improvements.

Each article included in this Research Topic not only advances our current understanding of AntibodyPlus therapeutics, but also stimulates further exploration into the diverse and evolving roles of antibody-based strategies in oncology. By integrating insights from molecular engineering, immunological mechanisms, and clinical applications, this collection encourages a more comprehensive and forward-looking perspective on the opportunities and challenges in antibody research. As the field continues to progress, it is increasingly evident that interdisciplinary collaboration and innovative technological approaches will be essential to fully realize the therapeutic potential of these versatile platforms. The studies presented here collectively highlight the pivotal role of AntibodyPlus therapeutics in shaping the future of cancer treatment, offering promising avenues for overcoming resistance, enhancing specificity, and improving patient outcomes.

The editorial team would like to express our sincere gratitude to all authors and reviewers for their valuable contributions to this Research Topic. We hope that the research compiled in this collection will serve as a useful resource for advancing the development of next-generation antibody therapeutics and will inspire continued innovation and collaboration within the scientific and clinical communities.

